# Identification and Characterization of a Ginsenoside-Transforming β-Glucosidase from *Pseudonocardia* sp. Gsoil 1536 and Its Application for Enhanced Production of Minor Ginsenoside Rg_2_(*S*)

**DOI:** 10.1371/journal.pone.0096914

**Published:** 2014-06-09

**Authors:** Juan Du, Chang-Hao Cui, Sung Chul Park, Jin-Kwang Kim, Hong-Shan Yu, Feng-Xie Jin, Changkai Sun, Sun-Chang Kim, Wan-Taek Im

**Affiliations:** 1 KAIST Institute for Biocentury, Korea Advanced Institute of Science and Technology, Yuseong-gu, Daejeon, Republic of Korea; 2 College of Biotechnology, Dalian Polytechnic University, Ganjingzi-qu, Dalian, P. R. China; 3 Department of Biological Sciences, Korea Advanced Institute of Science and Technology, Yuseong-gu, Daejeon, Republic of Korea; 4 Intelligent Synthetic Biology Center, Yuseong-gu, Daejeon, Republic of Korea; 5 Institute for Brain Disorders, Dalian Medical University, Dalian, P.R. China; 6 Department of Biotechnology, Hankyoung National University, Chungang-no Anseong-si, Republic of Korea; Centro de Biología Molecular Severo Ochoa (CSIC-UAM), Spain

## Abstract

The ginsenoside Rg_2_(*S*), which is one of the pharmaceutical components of ginseng, is known to have neuroprotective, anti-inflammation, and anti-diabetic effects. However, the usage of ginsenoside Rg_2_(*S*) is restricted owing to the small amounts found in white and red ginseng. To enhance the production of ginsenoside Rg_2_(*S*) as a 100 gram unit with high specificity, yield, and purity, an enzymatic bioconversion method was developed to adopt the recombinant glycoside hydrolase (BglPC28), which is a ginsenoside-transforming recombinant β-glucosidase from *Pseudonocardia* sp. strain Gsoil 1536. The gene, termed *bglPC28*, encoding β-glucosidase (BglPC28) belonging to the glycoside hydrolase family 3 was cloned. *bglPC28* consists of 2,232 bp (743 amino acid residues) with a predicted molecular mass of 78,975 Da. This enzyme was overexpressed in *Escherichia coli* BL21(DE3) using a GST-fused pGEX 4T-1 vector system. The optimum conditions of the recombinant BglPC28 were pH 7.0 and 37°C. BglPC28 can effectively transform the ginsenoside Re to Rg_2_(*S*); the *K*_m_ values of PNPG and Re were 6.36±1.10 and 1.42±0.13 mM, respectively, and the *V*_max_ values were 40.0±2.55 and 5.62±0.21 µmol min^−1 ^mg^−1^ of protein, respectively. A scaled-up biotransformation reaction was performed in a 10 L jar fermenter at pH 7.0 and 30°C for 12 hours with a concentration of 20 mg/ml of ginsenoside Re from American ginseng roots. Finally, 113 g of Rg_2_(*S*) was produced from 150 g of Re with 84.0±1.1% chromatographic purity. These results suggest that this enzymatic method could be usefully exploited in the preparation of ginsenoside Rg_2_(*S*) in the cosmetics, functional food, and pharmaceutical industries.

## Introduction

**Figure 1:**
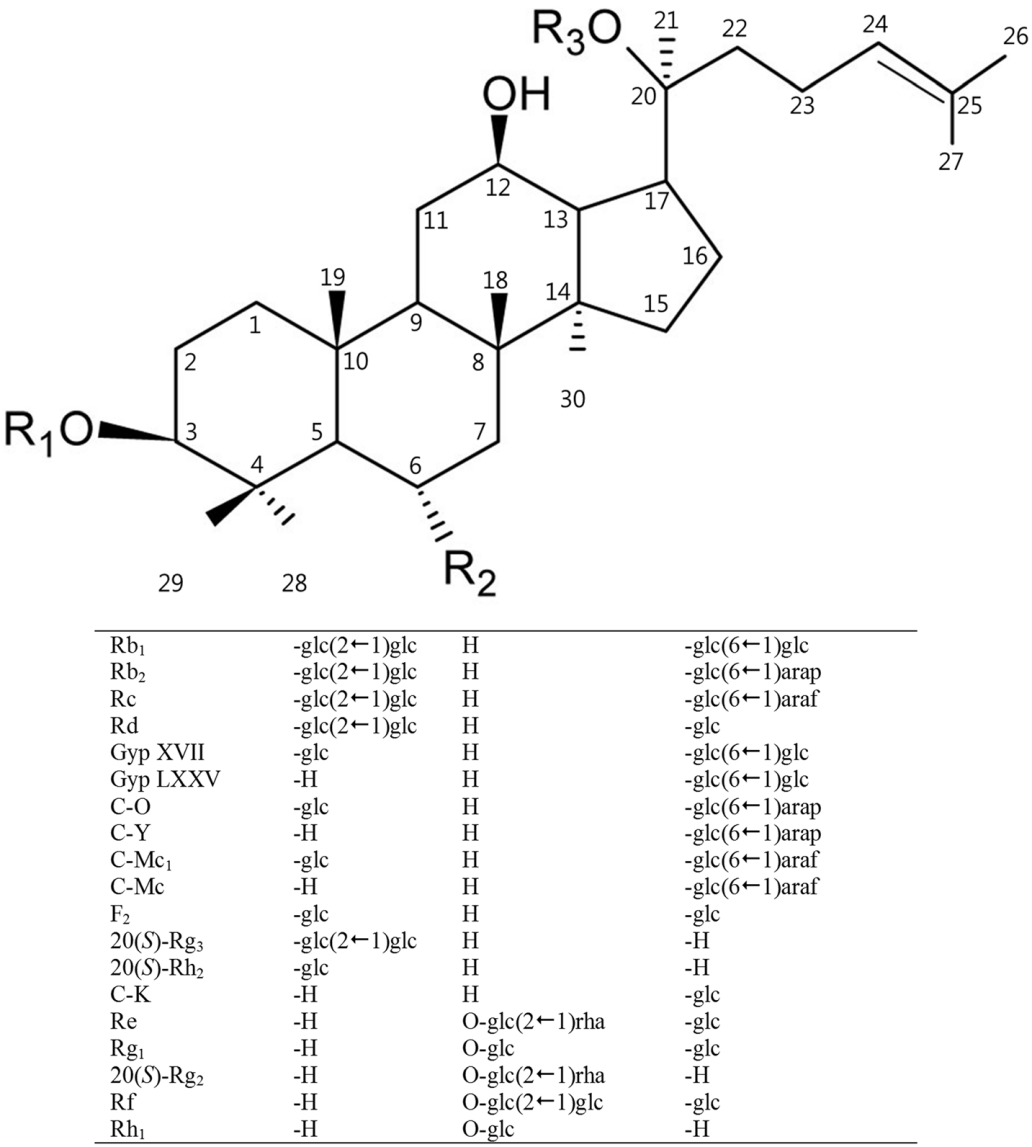
Chemical structures of protopanaxadiol and protopanaxatriol ginsenosides. The ginsenosides represented here are all (*S*)-type ginsenosides. glc, β-D-glucopyranosyl; arap, α-L-arabinopyranosyl; araf, α-L-arabinofuranosyl; rha, α-L-rhamnopyranosyl; Gyp, gypenoside; C, compound; C-O, compound O; C-Y, compound Y, C-Mc_1_, compound Mc_1_; C-Mc, compound Mc; C-K, compound K.

Ginseng, an important herbal medicine, has been widely used for thousands of years in East Asia and has been popularized in the West during the past decades [1], [2]. Reports show that ginseng has a range of pharmacological and therapeutic uses [3], [4], [5], [6], [7]. The ginseng root consists of ginsenosides, polysaccharides, peptide, polyacetylenic alcohols, and fatty acids [8]. Ginsenosides are the major active components of ginseng, and they appear to be responsible for the principle pharmacological activities of ginseng, including vasorelaxation and anti-neoplastic, anti-diabetic, anti-inflammation, and anti-oxidant effects [9], [10], [11]. Ginsenosides can be categorized as protopanaxadiol (PPD), protopanaxatriol (PPT), and oleanane saponins, based on the structure of the aglycon, with a dammarane skeleton [12]. The PPD- and PPT-type ginsenosides are further classified into subgroups based on the position and number of sugar moieties attached to the aglycon at positions C3 or C6 and C20. For example, one of the largest PPD-type ginsenosides, Rb_1_, contains 4 glucose moieties, with two attached via glycosidic linkages to the C3 and C20 positions, respectively, of the aglycon (Fig. 1). They may also be classified as major or minor ginsenosides based on the amount found in cultivated ginseng.

Ginsenosides Rb_1_, Rb_2_, Rc, Rd, Re, and Rg_1_ are major ginsenosides thatmake up more than 90% of total ginseng ginsenosides [3], [12]. Minor ginsenosides (Rg_3_, Rh_2_, F_2_, C-K, Rg_2_, Rh_1_, and F_1_) that are deglycosylated from the major ginsenosides exist in smaller amounts. As secondary metabolite compounds, the minor ginsenosides have some chemical reactivity that the major ginsenosides do not. Furthermore, there is emerging evidence that the minor ginsenosides have more important pharmaceutical effects, such as anti-cancer, anti-diabetic, anti-oxidative, and anti-aging effects, than the glycosylated major ginsenosides [10], [11], [13], [14], [15].

20(*S*)-GinsenosideRg_2_ [hereafter Rg_2_(*S*)] is a native ginsenoside found in Korean white and red ginseng (*Panax ginseng*, C.A. Meyer), and accounts forless than 0.02% of dried mass in red ginseng (a heat-treated ginseng with more minor ginsenosides) [16]. Ginsenoside Rg_2_(*S*) has significant neuroprotective pharmaceutical effects. It inhibits mitochondrial permeability transition pores in rat brain as a neuroprotective agent with mechanisms of anti-oxidation and anti-apoptosis [17]. Rg_2_ could hence potentially be used in a treatment strategy of Alzheimer’s disease [18]. Second, Rg_2_ improved neurological performance and memory ability of vascular dementia rats [19]. Other investigations suggest that Rg_2_ can protect cells against UVB-induced genotoxicityby increasing DNA repair and decreasing apoptosis [20], [21]. Rg_2_ was noted to have a significant impacton improving hemodynamic state and activity SOD (superoxide dismutase) in hemorrhagic shock dogs [22]. It was further reported that Rg_2_ inhibits leukocyte adhesion on the vascular wall in human cells, providing protection against vascular inflammatory disease [23]. Finally, Rg_2_ inhibits hepatic glucose production and showed potential for therapeutic treatment for type 2 diabetic patients [24].

Rg_2_(*S*) can be easily produced through a weakacid/high temperature process, i.e. the Korean red ginseng process. However, this process creates by products such as Rg_2_(*R*), Rg_4_, and Rg_6_. Rg_2_(*S*) consequently has low concentrations in Korean red ginseng or red ginseng extract [16], [25], [26]. Another method of obtaining Rg_2_(*S*) is to use microbes or enzymatic processes. One example is *Mucilaginibacter composti* TR6-03^T^, which has β-glucosidase activity and showed an ability to convert ginsenoside Re into Rg_2_(*S*) [27]. Although several ginsenoside hydrolyzing recombinant enzymes have been constructed, the majority of these have hydrolysis ability with respect to protopanaxadiol type ginsenosides (i.e. Rb_1_, Rb_2_, Rc, Rd) rather than protopanaxatriol type ginsenosides (i.e. Re, Rf, Rg_1_) [28], [29], [30], [31], [32], [33], [34], [35], [36]. Among these, three enzymes were reported to have hydrolysis activity for the glucose moiety at the C20 position of PPT aglycon [28], [29], [35]. However, Cui et al [28] and Quanet al [35] only conducted a simple enzyme characterization, without further scale-up or process engineering. Cui et al [29] also conducted enzyme characterization and subsequently obtained a gram scale Rg_2_(*S*) using a PPT type ginsenoside mixture. However, the yield was low (33.4%) and the final purification step using silica resin is complicated.

The experiment in this study was designed to overcome the above disadvantagesand to meet the industrial demand for mass production of ginsenoside Rg_2_(*S*) and fulfill the original purpose of its application as are combinant enzyme. From a dozen bacterial strains capable of transforming ginsenosides, we selected *Pseudonocardia* sp. Gsoil 1536 on the basis of its high activity for transforming Re into Rg_2_(*S*). A genome fosmid library was constructed, the key ginsenoside-transforming β-glucosidase (BglPC28) was identified, its novel gene was cloned for production as a recombinant enzyme, and the enzymatic properties and substrate specificities of the recombinant enzyme were thoroughly investigated. Treatment of purified ginsenoside Re with BglPC28 followed by purification yielded 100 gram-scale Rg_2_(*S*) with high purity. This is the first report of the 100 gram-scale production of high-purity Rg_2_(*S*) by the application of a recombinant glycoside hydrolase.

## Materials and Methods

### 2.1. Materials

Theginsenoside Re (purity: 87.6%) from the root of *Panax quinquefolius* from FusongBiotech Co. Ltd. (China) was used as the substrate in the currentinvestigation. Ginsenosides standards which are over 98% purity such as Rb_1_, Rb_2_, Rc, Rd, Rg_2_(*S*), Rh_2_(*S*), F_2_, compound K (C-K), protopanaxadiol (PPD), Rg_1_, Re, Rg_2_(*S*), Rh_1_(*S*), and protopanaxatriol (PPT) were purchased from Nanjing Zelang Medical Technology Co. Ltd. (China). Methanol and acetonitrile with HPLC grade were obtained from Merck (Darmstadt, Germany). The other chemicals used in this study were a minimum of analytical reagent grade, and the sources are noted individually in the Methods section. *Pseudonocardia* sp. Gsoil 1536, which has ginsenoside-hydrolyzing activity, was isolated from the soil sample in ginseng filed, Pocheon Province, South Korea, and cultivated on R2A agar (BD, USA) under aerobic conditions at 30°C and used for the gene cloning experiment. *Escherichia coli* BL21 (DE3) and pGEX 4T-1 plasmid (GE Healthcare, USA) were used as host, and expression vectorsources, respectively. The recombinant *E. coli* for protein expression was cultivated in a Luria-Bertani (LB) medium supplemented with ampicillin (100 mg/l).

### 2.2. Fosmid Library Construction and Fosmid Sequencing

A CopyControl™ Fosmid Production kit (Epicentre Technologies, WI) was used to clone the ginsenoside hydrolyzing glycosidase gene from *Pseudonocardia* sp. Gsoil 1536. A fosmid library was constructed according to the manufacturer’s protocol. Infected *E. coli* was transferred onto LB plates supplemented with 40 µg/ml X-Glc (5-bromo-4-chloro-3-indolyl β-D-glucopyranoside) and 12.5 µg/ml chloramphenicol and then incubated at 37°C for 16 h. The blue color clones were selected as putative ginsenoside hydrolyzing clones. After confirmation of the ginsenoside-hydrolyzing activity by a TLC assay, one clone was selected for fosmid sequencing. Fosmid DNA was purified according to the manufacturer’s protocols (Fosmid MAX DNA purification kit, Epicentre, WI) and was sequenced by Macrogen Co. Ltd. (Korea). The final sequences assembly procedure was conducted by the SeqMan program in the DNASTAR package (DNASTAR, WI), which yieldedtwo contigs (14.3 - and 4.7 kb).

### 2.3. Phylogenetic Analysis of BglPC28

Database homology search was performed with BLAST programprovided by NCBI. Sequences of the characterized glycosyl hydrolases were obtained from the CAZY database [Carbohydrate-Active enZymes database (http://www.cazy.org)], and multiple alignments were performed using the CLUSTAL_X program [37]. Gaps were edited in the BioEdit program [38], and evolutionary distances were calculated using the Kimura two-parameter model [39]. A phylogenetic tree was constructed using the neighbor-joining method [40] in the MEGA5 Program [41], with bootstrap values based on 1000 replicates [42]. Furthermore, the multiple amino acid sequence alignment and the conserved patterns of discrete amino acid sequences of BglPC28 and known the most homologousβ-glucosidases were performed by using ClustalW2 program (http://www.ebi.ac.uk/Tools/msa/clustalw2/).

### 2.4. Molecular Cloning, Expression, and Purification of Recombinant BglPC28

The assembled DNA sequence was analyzed using the ORF Finder program on the NCBI website (www.ncbi.nlm.nih.gov/gorf). Predicted ORFs were subjected to a similarity search using BLASTP, which identified two putative open reading frames of a β-glucosidase belonging to glycosyl hydrolase family 3. The sequence of the oligonucleotide primers used for gene cloning was based on the DNA sequence of *bglPC28* (GenBank accession no. JX960416). Forward (5′- GGTTCCGCGTGGATCCATCGACCCCGTTGATCTCACCCTC-3′) and reverse primers (5′- GATGCGGCCGCTCGAGCTAGTTTTGTCCGACGTGTTGGGG-3′) were designed as the primers to introduce the *Bam*HI and *Xho*I restriction sites (underline), respectively, and were synthesized by BioneerCo. Ltd. (Daejeon, Korea). The amplified DNA fragment obtained from the PCR was purified and insertedinto the pGEX 4T-1GST fusion vector digested with *Bam*HI and *Xho*I using an EzCloning Kit (Enzynomics Co. Ltd., Korea). The resulting recombinant pGEX-*bglPC28* was transformed into *E. coli* BL21(DE3). The *E. coli* BL21(DE3) harboring the recombinant plasmid was grown in an LB-ampicillin medium at 37°C until the culture reached an OD_600_ of 0.6, at which point the protein expression was induced through the addition of 0.1 mM isopropyl-β-D-thiogalactopyranoside (IPTG). The bacterial cells were incubated for a further 24 h at 22°C and were then harvested via centrifugation at 13,000 rpm for 15 min at 4°C. The cells were washed twice with a solution consisting of 100 mM sodium phosphate and 1% Triton X-100 (pH 7.0); then, they were resuspended in 100 mM sodium phosphate (pH 7.0). The cells were disrupted via ultrasonication (Vibra-cell, Sonics & Materials, CT, USA). The intact cells and debris were removed via centrifugation at 13,000 rpmfor 15 min at 4°C in order to obtain the crude cell extract. The GST tag was purified using the GST bind agarose resin (Elpisbiotech Co. Ltd, Korea). The homogeneity of the protein was assessed using 10% SDS-PAGE and an EZ-Gel staining solution (Daeillab Co. Ltd., Korea).

### 2.5. Effect of pH, Temperature, Metal Ions and Chemical Reagent on Enzyme Activity

The specific activity of purified BglPC28 was determined using p-nitrophenyl-β-D-glucopyranoside (pNPG) as a surrogate substrate in 50 mM sodium phosphate buffer, pH 7.0 at 37°C. Reactions were stopped after 10 minutes (min) by the addition of Na_2_CO_3_ at a final concentration of 0.5 M, and the release of p-nitrophenol was measured immediately using a microplate reader at 405 nm (Bio-Rad model 680; Bio-Rad, Hercules, CA). One unit of activity was defined as the amount of protein required to generate 1 µmol of p-nitrophenol per min. Specific activity was expressed as units per milligram of protein. Protein concentrations were determined using the bicinchoninic acid (BCA) protein assay (Pierce, Rockford, IL), with bovine serum albumin (Sigma) as the standard. All assays were performed in triplicate.

The effect of pH on enzymatic activity was determined using 1.0 Mm pNPG as a substrate in the following buffers (each at 50 mM): KCl-HCl (pH 2.0), glycine-HCl (pH 3.0), sodium acetate (pH 4.0 and 5.0), sodium phosphate (pH 6.0, 7.0 and 7.5), Tris-HCl (pH 8.0, and 9.0) and glycine-sodium hydroxide (pH 10). The pH stability of recombinant BglPC28 was determined by measuring enzymatic activity after incubation in each buffer (containing 2.0 mM pNPG in 50 mM potassium buffer as a substrate) for 12 h at 4°C. The results are expressed as a percentage of the activity obtained at the optimum pH. The effect of temperature on enzymatic activity was tested by incubating the enzyme at various temperatures ranging from 4 to 65°C (4, 10, 18, 25, 30, 37, 45, 55, 65°C) at optimum pH for 5 min in 50 mM potassium phosphate buffer containing 2.0 mM pNPG. The thermo-stability of the enzyme was examined by incubating the enzyme in 50 mM potassium phosphate buffer for 30 min at different temperatures. After cooling the sample on ice for 10 min, activity was determined using pNPG as the substrate.

The effects of metals and other chemicals on BglPC28 activity were also determined. BglPC28 activity was tested in the presence of 1 or 10 mM (final concentration) of HgCl_2_, MnCl_2_, CaCl_2_, CoCl_2_, MgCl_2_, EDTA, NaCl, KCl, CuCl_2_, SDS, dithiothreitol (DTT), or β-mercaptoethanol for 30 min at 37°C. The remaining activity was determined using pNPG as a substrate, and activities are expressed as a percentage of the activity obtained in the absence of the compound.

Substrate preference was examined using 2.0 mM chromogenic o-nitrophenyl (ONP) and p-nitrophenyl (PNP) as substrates at 37°C for 5 min, with one activity unit being defined as the release of 1 µmol o-nitrophenol or p-nitrophenol per min. The following substrates were tested: PNP-β-D-glucopyranoside, PNP-β-D-galactopyranoside, PNP-β-D-fucopyranoside, PNP-N-acetyl-β-D-glucosaminide, PNP-β-L-arabinopyranoside, PNP-β-D-mannopyranoside, PNP-β-D-xylopyranoside, PNP-α-D-glucopyranoside, PNP-α-L-arabinofuranoside, PNP-α-L-arabinopyranoside, PNP-α-L-rhamnopyranoside, PNP-α-D-mannopyranoside, PNP-α-D-xylopyranoside, ONP-β-D-glucopyranoside, ONP-β-D-galactopyranoside, ONP-β-D-fucopyranoside and ONP-α-D-galactopyranoside (all from Sigma).

### 2.6. Determination of Kinetic Parameters

Kinetic studies were performed with freshly purified enzymes using pNPG at 1–20 mM and Re at concentrations from 0.2 mM to 5.0 mM. One unit of activity was defined as the amount of protein required to generate 1 µmol of p-nitrophenol or to convert 1 µmol of Re per minute. All assays were performed in triplicate. The parameters, *K*_m_ and *V*_max_, were determined using the enzyme kinetics program described by Cleland [43].

### 2.7. Biotransformation Activity of Ginsenosides Using BglPC28

The initial biotransformation experiments using the major ginsenosides Rb_1_ and Re as substrates revealed that the GST-fused enzyme does not affect the activities of BglPC28. Therefore, the fusion protein (GST-BglPC28) was used to determine the specificity and selectivity of the enzymes for the hydrolysis of the glucose moieties attached at the C3 and C20 positions in the five PPD ginsenosides. The enzyme solutions at a concentration of 0.1 mg/ml in 100 mM of sodium phosphate buffer (pH 7.0) were reacted with an equal volume of Rb_1_, Rb_2_, Rb_3_, Rc, Rd, Re, and Rg_1_ solution at a concentration of 0.2% (w/v) in 100 mM of sodium phosphate buffer (pH 7.0) at 37°C. The samples were taken at regular intervalsand analyzed via TLC or HPLC after pretreatment (see analytic methods).

### 2.8. Optimization of Concentration of the Enzyme and Substrate

In order to determine the optimal condition for the biotransformation of Re to Rg_2_(*S*), the substrate concentration of Re in the reaction was optimized. The final crude BglPC28 concentration was fixed to 10, 20, and 50 mg/ml and reacted with ginsenoside Redissolved in DMSO (100 mg/ml) in order to have 20 and 30 mg/ml as the final substrate concentration. These 6 types of optimization reactions were performed in a 2 ml eppendorf tube with a 1 ml working volume at 200 rpm for 24 h at 37°C. The samples were taken at regular intervals and analyzed via TLC and HPLC.

### 2.9. Preparation of the Recombinant Enzymes BglPC28 using High Cell Density Culture

For the production of the recombinant BglPC28, the LB medium supplemented with ampicillin (100 µg/ml) was used to cultivate the *E. coli* harboring pGEX-*bglPC28* in a 10 L stirred-tank reactor (BiotronGX, Hanil science Co., Korea) with a 5 L working volume at 500 rpm. The pH value of the medium was adjusted to 7.0 using 100 mM of sodium phosphate buffer. The culture was incubated at 37°C until the culture reached an OD of 3.0 at 600 nm. The protein expression was induced through the addition of isopropyl-β-D-thiogalactopyranoside (IPTG) with a final concentration of 0.1 mM with feeding 2% (w/v) glucose. The bacterial cells were incubated for a further 18 h at 22°C and were then harvested via centrifugation at 5,000 rpm for 20 min (Component R, Hanil science Co Ltd., Korea) at 4°C.

The cells suspended in 100 mM of phosphate buffer (pH 7.0) were disrupted via sonication (Vibra-cell, Sonics & Materials, CT, USA), and then the intact cells and debris were removed via centrifugation at 5,000 rpm for 20 min (Component R, Hanil science Co Ltd., Korea) at 4°Cin order to obtain the supernatants of the crude enzymes. The crude recombinant BglPC28 was diluted to the desired concentration with 100 mM of sodium phosphate buffer (pH 7.0) and was used to biotransform the Re.

### 2.10. Scaled-up Biotransformation of Ginsenoside Re via Crude BglPC28

The scaled-up biotransformation was performed in a 10 L stirred-tank reactor (BiotronGX, Hanil science Co., Korea) with a 7.5 L working volumecontaining 10% DMSO (Dimethyl sulfoxide) at 100 rpm for 24 h. The reaction was performed under conditions in pH 7.0 at 30°C. The reaction started with a composition of 20 mg/ml of substrate ginsenoside (Re; total 150 g) as final concentration and 1.5 L of crude recombinant BglPC28 (20 mg/ml) in 100 mM of phosphate buffer (pH 7.0). Samples were collected at regular intervalsand were analyzed by HPLC in order to determine the production of the ginsenoside Rg_2_(*S*) from Re.

### 2.11. Purification of Rg_2_(*S*)

Following the 7.5 L reaction of Re with BglPC28, the mixture was cooled at 4°C and centrifuged at 5,000 rpm for 15 min (Component R, Hanil science Co Ltd., Korea). The biotransformed ginsenoside Rg_2_(*S*) in the supernatants and precipitates was processed separatelyin order to purify the samples. The precipitate was also dissolved in 5.0 L of 70% ethanol solution twice and filtered through a filter paper (Advantec, Japan). The ethanol extracts were combinedand adjusted to be a 40% ethanol solution. The column chromatography [3,170(L)×128(D) mm; Doointech, Korea] packed with HP20 resin (Mitsubishi, Japan) was adopted in order to remove the impurities, except the ginsenosides. The supernatants and 40% ethanol solution were loaded on to the column together. The free sugar molecules and unwanted hydrophilic compounds from the HP-20 that were adsorbed in beads were washed with 6 bed volumes (BV) of water, and finally the adsorbed ginsenosides were eluted using 6 BV of 95% ethanol (extra pure grade; SK Chemicals, Korea). The ethanol eluent was evaporated *in vacuo*. The resulting powder was dissolved in 100% methanol and analyzed via HPLC.

### 2.12. Analytic Methods

#### 2.12.1. Thin Layer Chromatography (TLC) Analysis

The thin layer chromatography (TLC) was performed using 60f_254_ silica gel plates (Merck, Germany) with Chcl_3_-CH_3_OH-H_2_O (65∶35∶10, lower phase) as the solvent. The spots on the TLC plates were identified through comparisons with standard ginsenoside after visualization was made by spraying 10% (vol/vol) H_2_SO_4_, followed by heating at 110°C for 5 min.

#### 2.12.2. High Performance Liquid Chromatography (HPLC) analysis

HPLC analysis of the ginsenosides [Rb_1_, Rd, Rg_2_(*S*), Rh_2_(*S*), F_2_, C-K, Rg_1_, Re and Rg_2_(*S*)] was performed using an HPLC system (Younglin Co., Ltd., Korea), with a quaternary pump, automatic injector, single-wavelength UV detector (model 730 D), and Younglin’s AutoChro 3000 software for peak identification and integration. The separation was carried out on a Prodigy ODS (2) C18 column (5 µm, 150×4.6-mm i.d.; Phenomenex, USA) with a guard column (5 µm, 12.5×4.6-mm i.d.; Eclipse XDB C18). The mobile phases were acetonitrile (A) and water (B). Gradient elution started with 17% solvent A and 83% solvent B changed to: A from 17 to 25%, 12–20 min; A from 25 to 32%, 20–30 min; A from 32 to 55%, 30–35 min; A from 55 to 60%, 35–40 min; A from 60 to 80%, 40–45 min; A from 80 to 100%, 45–50 min; A 100%, 50–54 min; A from 100 to 17%, 54.0–54.1 min; and A 17%, 54.1–65 min. The flow rate was 1.0 ml/min; detection was performed by monitoring the absorbance at 203 nm and an injection volume of 25 µl.

#### 2.12.3. LC/MS/MS analysis

Electrospray ionization mass spectrum (ESI-MS) was measured for the biotransformed Rg_2_(*S*) after purification steps on a triple-quadrupole tandemmass spectrometer (API-2000, Applied Biosystems, Foster City, USA) with negative ion mode. The ESI parameters were as follows: ionspray voltage, −4,200 V; ion source gas 1 (GS1), 20; curtain gas (CUR), 20; collision gas (CAD), 2. The declustering potential (DP), focusing potential (FP), entrance potential (EP), collision cell exit potential (CXP) and collision energy (CE) were variant with regard to measured ginsenosides. For full-scan MS analysis, the spectra were recorded in the m/z range from 400 to 1,000.

## Results

### 3.1. Fosmid Library Construction and Cloning of BglPC28

β-Glucosidase activity was detected from a ginsenoside-hydrolyzing bacterium, *Pseudonocardia*sp. strain Gsoil 1536, isolated from the soil of a ginseng cultivating field. Since strain Gsoil 1536 converted ginsenosides Rb_1_ and Rd into C-K via F_2_ as well as converting Re and Rg_1_ into Rg_2_(*S*) and Rg_1_(*S*), respectively (data not shown), it could have two types of β-D-glucosidase activities toward β-1,2- and β-1,6-glucosidic linkages. To isolate and clone the respective genes for the ginsenoside-hydrolyzing enzymes from strain Gsoil 1536, we created a fosmid library, screened a clone containing the gene encoding the enzymatic activity, and determined the two contigs (14.3 - and 4.7 kb) partial sequence of the fosmid vector. An ORF FINDER (www.ncbi.nlm.nih.gov/gorf) analysis of the large contig of the 14.3 kb revealed that it encoded a total of 11 putative ORFs of longer than 300 codons (data not shown). Four putative ORFs of longer than 300 codons (data not shown) were revealed in the small size contig of 4.7 kb. Among three ORFs, one was homologousto glycoside hydrolase genes in glycoside hydrolase family 3(GH3). This ORF, termed *bglPC28*, consisting of 2,232 bp encoding 743 amino acids, was amplified via PCR and then inserted into the pGEX 4T-1 vector.

### 3.2. Phylogenetic Analysis of BglPC28 Sequences

**Figure 2:**
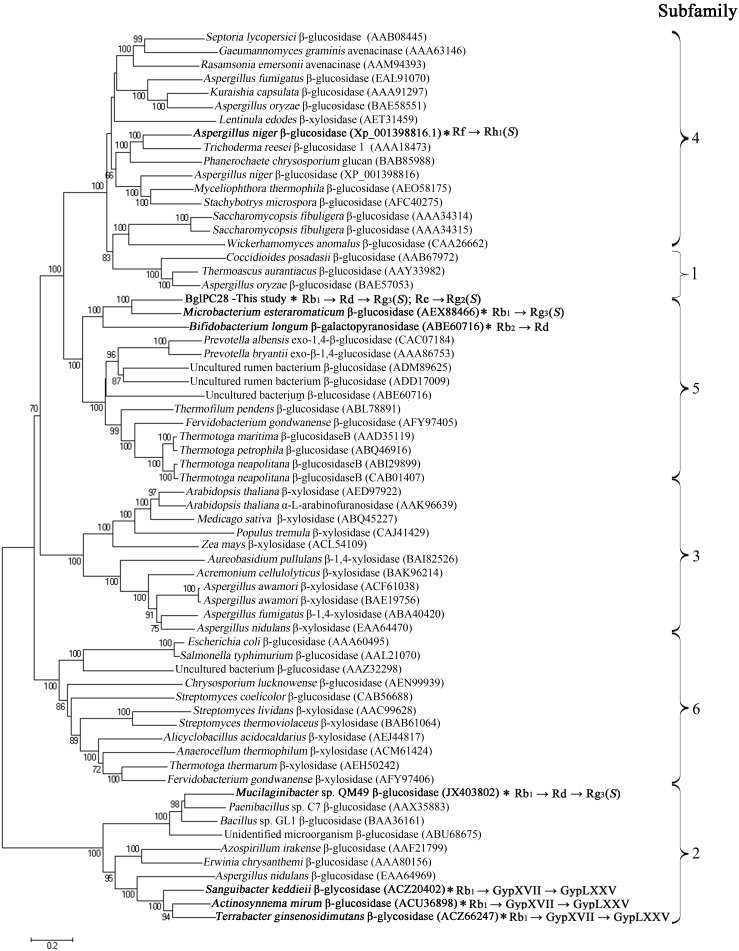
Phylogenetic analysis of characterized glycoside hydrolases family 3 (GH3). Amino acid sequences were obtained from the NCBI/EMBL database and CAZy database (accession numbers are indicated on the tree). This tree was made using the neighbor-joining method with a poisson model and pairwise deletion. Bootstrap values expressed as percentages of 1,000 replications greater than 65% are shown at the branch points. The bar represents 20 amino acid residues substitutions per 100 amino acid residues.

Analysis of the amino acid sequences of BglPC28 indicated that it was 61.7% identical to the glycoside hydrolase (Bgp1) of *Microbacterium esteraromaticum* KACC 16318 (GenBank number AEX88466), which belongs to GH3. The enzymatic activity of Bgp1 has been characterized to have ginsenoside conversion activity [35]. BglPC28 has homology to the protein domain of GH3. The Carbohydrate-Active enZymes database (http://www.cazy.org) describes more than 5,000 uncharacterized and 222 characterized GH3 members that are widespread across numerous organisms. Glycosyl hydrolases are classified according to their amino acid sequence similarities, which reflect the structural features and substrate specificities of the enzymes (http://www.cazy.org/fam/acc_GH.html). The GH3 is subdivided into six subfamilies [44]. In order to determine the evolutionary position of BglPC28 within the characterized enzymes in glycoside hydrolasesfamily 3, a phylogenetic analysis was conducted using the neighbor-joining method in the MEGA5 Program with bootstrap values based on 1,000 replications. The resulting consensus tree is presented in Fig. 2. BglPC28 clustered within subfamily 5 and formed a separate, well-supported clade (bootstrap of 100) with β-glucosidase (Bgp1) derived from *Microbacterium esteraromaticum* KACC 16318, which was characterized. Several ginsenoside-hydrolyzing β-glucosidases in GH3 have previously been cloned, including a β-glucosidase (rApy-H11) from *Bifidobacterium longum* H-1 [45], a β-glucosidase (BgpA) from *Terrabacterginsenosidimutans* [46], a β-glucosidase (BGL1) from *Aspergillusniger* [47], a β-glucosidase (BglAm) from *Actinosynnemamirum* [28], a β-glucosidase (BglQM) from *Mucilaginibacter* sp. QM49 [29], a β-glucosidase (BglSk) from *Sanguibacter keddieii* [31], and a β-glucosidase (BglBX10) from *Flavobacterium johnsoniae*UW101^T^ [48]. The relationship between BglPC28 and these ginsenoside-hydrolyzing β-glucosidases is presented in Fig. 2.

**Figure 3:**
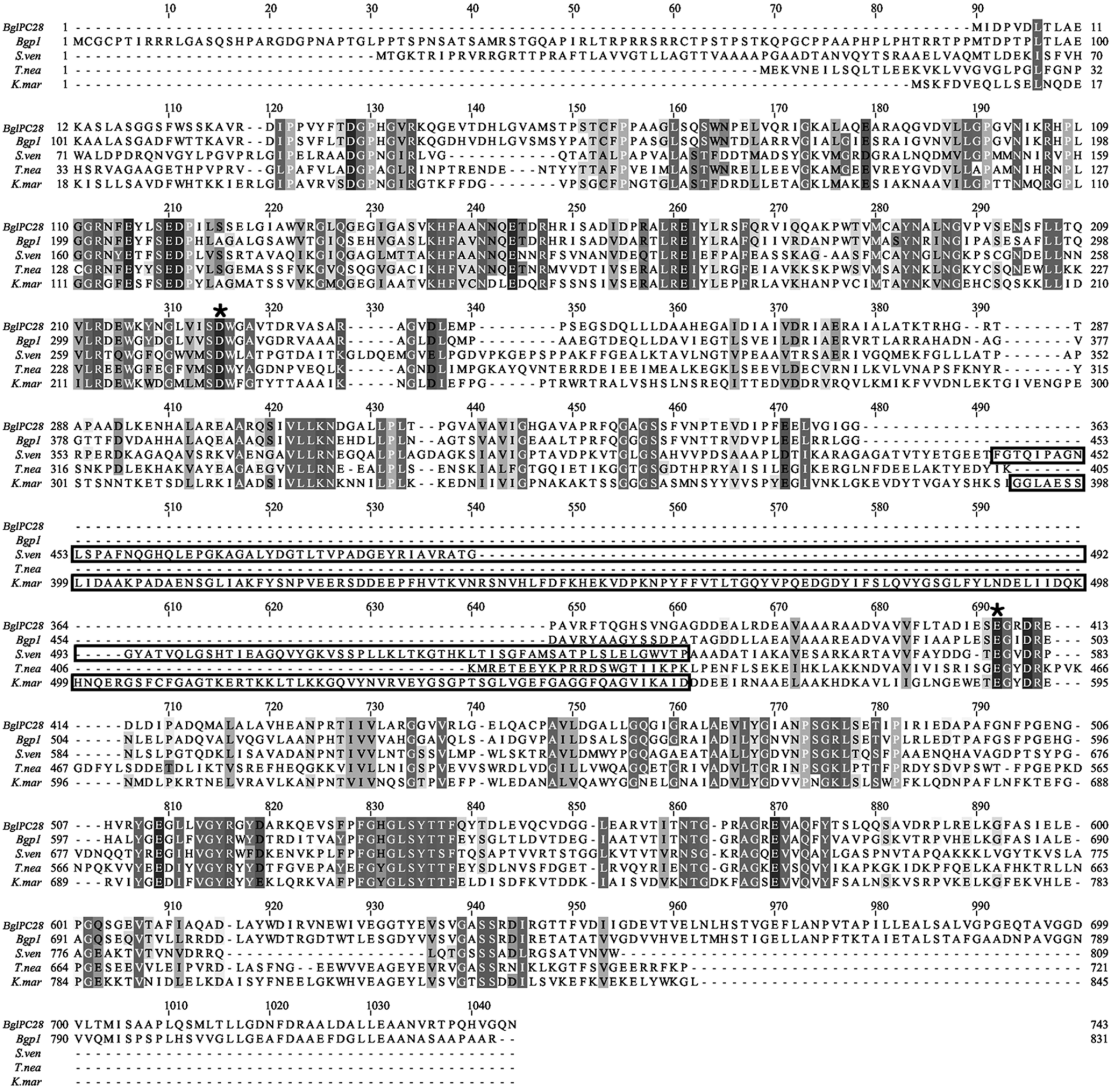
Sequence alignment of ginsenoside-transforming BglPC28 and Bgp1 or structure-determined glycoside hydrolase family 3 enzymes. The sequence alignment was created using ClustalW at the EBI-server using default settings and visualized with Jalview (http://www.ebi.ac.uk/Vmichele/jalview/). Regions of identity or high similarity among sequences are shown as black or gray columns, respectively. The conserved active site residues (general acid/base and nucleophile residues) were marked by asterisk. The PA14 domains of *S.ven* and *K.mar* are boxed. Genbank IDs of the glycoside hydrolases family 3 are as follows, *Pseudonocardia* sp. Gsoil 1536 β-glucosidase [*BglPC28* (This study)], JX960416; *Microbacterium esteraromaticum* KACC 16318 β-glucosidase (*Bgp1*), AEX88466; *Streptomyces venezuelae* β-glycosidase (*S.ven*), AAC68679; *Thermotoganeapolitana*DSM 4359^T^ β-glycosidase (*T.nea*), ABI29899; *Kluyveromycesmarxianus* β-glucosidase (*K.mar*), ACY95404.

Comparison of the BglPC28 sequence with ginsenoside-transforming enzyme (Bgp1) from *Microbacterium esteraromaticum* [35] and known-structure β-glucosidases from *Streptomyces venezuelae* [49], *Thermotoganeapolitana* [50] and *Kluyveromycesmarxianus* [51] are shows in Fig. 3. The amino acids - D242 and E408 of BglPC28 which were marked by asterisk were thought to serve as nucleophile and the acid–base during the hydrolysis reaction through sequence alignment with the structure-determined above mentioned three β-glucosidases. The PA14 domain which is existed in many proteins such as glycosidases, glycosyl-transferases, proteases, amidases, toxins, adhesins and signaling molecules were found in some GH3 enzymes [52]. PA14 domain was found 230 of analyzed 820 sequences of GH3, and may have a carbohydrate-binding or fundamental role in biological events [51]. However, no PA14 domain was found in BglPC28 and Bgp1.

### 3.3. Expression and Purification of Recombinant BglPC28

**Figure 4:**
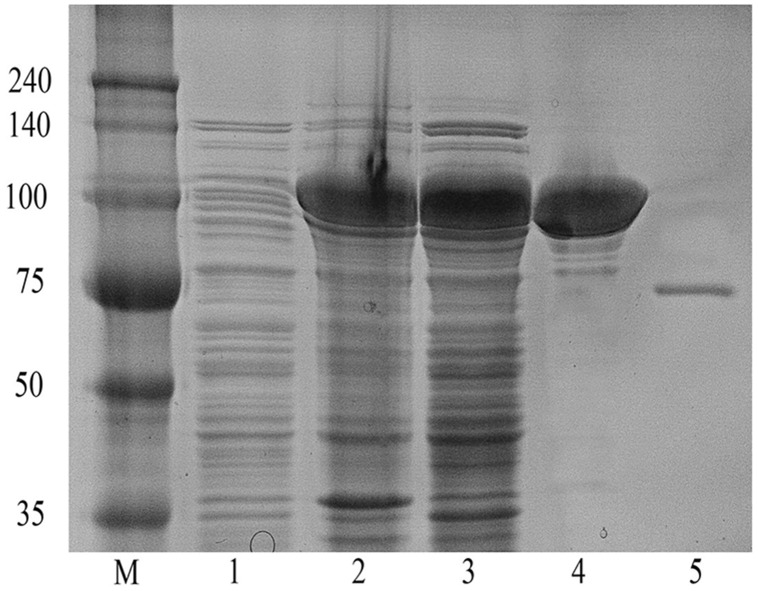
SDS-PAGE analysis of the recombinant BglPC28 after purification using the GST•bind agarose resin. Lane: 1, uninduced crude extract; 2, soluble fraction of the crude extract of the induced recombinant BL21 (DE3) cells; 3, precipitated fraction of the crude extract of the induced recombinant BL21 (DE3) cells; 4, GST-BglPC28 after purification with the GST**•**bind agarose resin; 5, purified recombinant BglPC28 after cleavage via thrombin.

In order to maximize the yield of the fusion protein in a soluble form, different induction conditions were tested and it was found that induction with 0.15 mM IPTG at 22°C for 18 h cultivation after initial induction produced the maximum level of soluble active fusion enzyme (data not shown). The GST-BglPC28 fusion protein was purified using the GST•bind agarose resin, and the GST tag was removed using thrombin at room temperature for 12 h incubation. The recombinant enzyme was purified by GST•bind agarose resin, and then supernatant from cell lysates as well as purified protein was applied to SDS-PAGE (Fig. 4). The molecular mass of the GST-BglPC28 calculated via an amino acid sequence was 105 KDa, which is similar mass detected in SDS-PAGE. In addition, the recombinant GST•BglPC28 contains 36.5±1.4% of total soluble protein in *E. coli* lysate. This high expression level in soluble form makes it more feasible for industrial application.

### 3.4. Characterization of Recombinant BglPm

**Figure 5:**
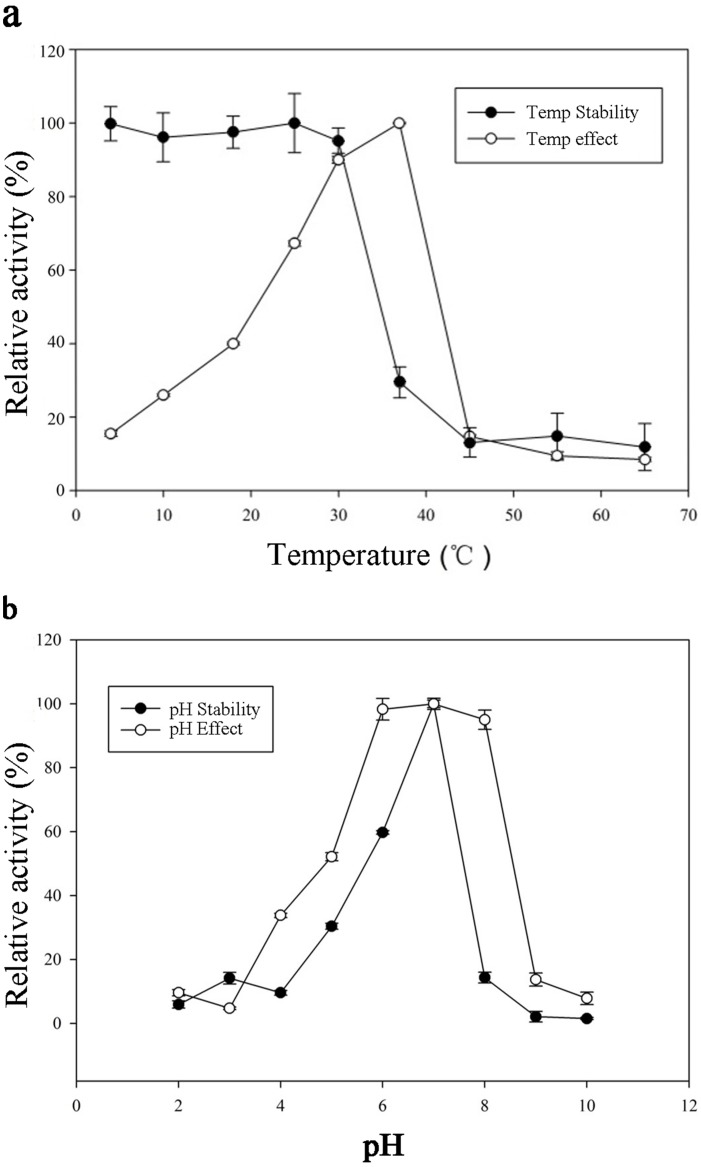
Effects of temperature (a) and pH (b) on the activity and stability of recombinant BglPC28.

The optimum temperature and pH were determined using purified GST-BglPC28. The optimal temperature activity of BglPC28 was 37°C; at 30°C and 25°C, the enzyme showed 90.0% and 67.2% relative activity, respectively, while thermostability decreased quickly beyond 37°C (Fig. 5a). The enzyme was stable at temperatures lower than 30°C, and about 70.6% of the activity was lost after incubation at 37°C for 30 min (Fig. 5b). BglPC28 had optimal pH activity and stability at pH 7.0 in a sodium phosphate buffer at 30°C; from pH 8.0, the enzyme stability decreased swiftly, while at pH 5.0 the enzyme activity decreased to 58.1% (Fig. 5b). The enzyme is probably mesophilic and stable at a neutral pH range. These optimal conditions are consistent with the soil environment from which *Pseudonocardia* sp. Gsoil 1536 was isolated. In addition, the near-neutral optimal pH and mild optimal temperature of BglPC28 are similar to those of other ginsenoside-hydrolyzing GH3 from bacteria [28], [29], [35], [46], [47]. Although the optimum temperature of BglPC28 for pNPG is 37°C, the ginsenoside-conversion reaction occurred at 30°C for extension of stable transformation activity.

The effects of metal ions, EDTA, β-mercaptoethanol, and SDS, on BglPC28 activity were also investigated (Table 1). The enzyme did not require Mg^2+^ for activity. Furthermore, it lost its activity in the presence of 50 mM Cu^2+^, and was significantly inhibited by 1 mM Hg^2+^ or 10 mM SDS. BglPC28 activity was not affected by DTT and β-mercaptoethanol, which are well-known thiol group inhibitors. These results suggest that sulfhydryl groups may not be involved in the catalytic center of the enzyme. The chelating agent EDTA did not inhibit BglPC28 activity, which indicated that divalent cations are not required for enzymatic activity. Thus, no dramatic positive effects on the activity of the BglPC28 were found for the tested ions (Table 1).

**Table 1:** Effects of metal ions and reagents on the activity of purified recombinant BglPC28.

Metal ions or regents	Relative activity ± SD (%) at:	
	1 mM	10 mM
NaCl	106.0±6.02	126.7±3.82
KCl	109.2±2.22	121.7±5.68
MgCl_2_	111.0±8.74	121.9±0.91
MnCl_2_	112.4±2.01	104.5±8.89
CoCl_2_	130.0±7.37	139.5±2.57
ZnCl_2_	110.1±2.69	124.4±4.59
CaCl_2_	111.3±1.71	123.3±3.13
CuCl_2_	103.3±6.46	108.4±7.71
HgCl_2_	1.1±0.534	2.0±0.74
SDS	0.9±0.540	1.1±0.81
EDTA	98.2±0.73	92.6±0.66
β-Mercaptoethanol	94.5±7.50	94.4±5.22
DTT	92.4±3.41	90.5±4.24
Control	100±5.02	100±6.03

The substrate specificity of BglPC28 was tested using 2.0 mM pNP- and oNP-glycosides with α and β configurations. The results, which are summarized in Table 2, showed that BglPC28 was maximally active against pNP-β-D-glucopyranoside, followed by pNP-β-L-arabinopyranoside, but had little effect on various other PNP- and ONP-glycosides, with the exception of pNP-α-D-glucopyranoside, pNP-β-D-glucosaminide, and pNP-β-D-xylopyranoside. The specific activity of BglPC28 against pNP-β-D-xylopyranoside is not seen in ginsenoside-transforming GH3 members from *Terrabacter ginsenosidimutans* [46], *Microbacterium esteraromaticum* [35], and *Mucilaginibacter* sp. QM49 [29].

**Table 2:** Relative activity of purified recombinant BglPC28 towards various chromogenic substrates as measured by PNP or PNP release at 37°C. ND: not determined.

Substrate^a^		Relative activity ± SD (%)^b^
1	pNP-α-D-glucopyranoside	7.6±2.4
2	pNP-α-D-mannopyranoside	ND
3	pNP-α-D-xylopyranoside	ND
4	pNP-α-L-arabinofuranoside	ND
5	pNP-α-L-arabinopyranoside	ND
6	pNP-α-L-rhamnopyranoside	ND
7	pNP-β-D-fucopyranoside	ND
8	pNP-β-D-galactopyranoside	ND
9	pNP-β-D-glucopyranoside	100.0±1.6
10	pNP-β-D-glucosaminide	7.3±5.5
11	pNP-β-D-mannopyranoside	ND
12	pNP-β-D-xylopyranoside	11.0±2.7
13	pNP-β-L-arabinopyranoside	14.5±3.6
14	oNP-α-D-galactopyranoside	ND
15	oNP-β-D-fucopyranoside	ND
16	oNP-β-D-galactopyranoside	ND
17	oNP-β-D-glucopyranoside	ND
18	pNP-α-L-D-fucopyranoside	ND

a Final concentration, 2.0 mM.

b Activity toward pNP-β-D-glucopyranoside was set as 100%.

### 3.5. Determination of Kinetic Parameters

The kinetic parameters of *V*_max_ and *K*_m_ of BglPC28 were determined by plotting the substrate concentration vs. the initial velocity of each reaction and subjecting the data to a linear regression analysis. The *K*_m_, *V*_max_, *k*_cat_, and *k*_cat_/*K*_m_ values for 4 substrates are presented in Table 3. The *K*_m_ values of BglPC28 for pNPG and Re were 6.36±1.10 and 1.42±0.13 mM, respectively, and the *V*_max_ values were 40.0±2.55 and 5.62±0.21 µmol min^−1 ^mg^−1^ of protein, respectively. The *k*_cat_ values were 52.7±3.4, and 7.40±0.28 S^−1^, respectively measured using the method described by Cleland [43].

**Table 3:** Kinetic parameters of recombinant BglPC28 on pNPG and ginsenoside Re.

Substrate	*K*_m_ (mM)	*V*_max_ (µmol min^−1^ mg^−1^)	*k*_cat_ (S^−1^)	*k*_cat_/*K*_m_ (mM^−1^ S^−1^)
pNPG	6.36±1.10	40.0±2.55	52.7±3.4	8.63±2.02
Re	1.42±0.13	5.62±0.21	7.40±0.28	5.28±0.69

The catalytic efficiencies (*k*_cat_/*K*_m_) for pNPG and Re decreased in this order: pNPG (8.63±2.02 mM^−1^ S^−1^) >Re (5.28±0.69 mM^−1^ S^−1^). The catalytic efficiencies for ginsenosides are higher than those of β-glycosidase from *Sulfolobus acidocaldarius* [53] for ginsenosides Rd (4.8 mM^−1^ min^−1^) and Rb_1_ (4.8 mM^−1^ min^−1^), and β-glucosidase from *Mucilaginibacter* sp. QM49 [29] for ginsenosides Rg_1_ (1.64±0.22 mM) and Re (0.51±0.07 mM).

### 3.6. Biotransformation Characteristics of BglPC28

**Figure 6:**
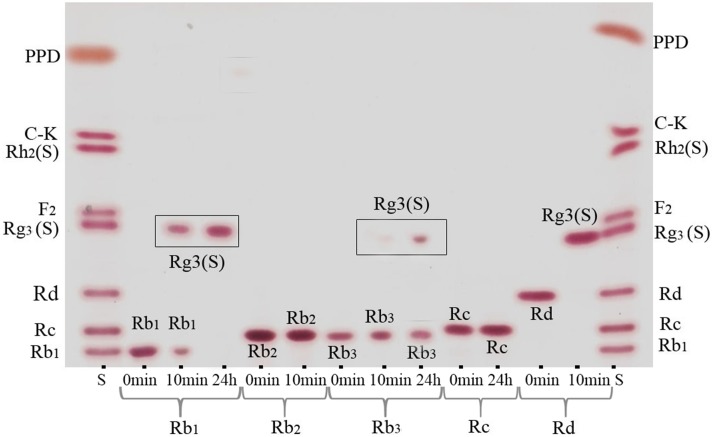
Thin layer chromatography (TLC) analyses of biotransformation of Rb_1_, Rb_2_, Rb_3_, Rc and Rd by recombinant BglPC28. The sampling times were 10: CHCl_3_-CH_3_OH-H_2_O (65∶35∶10, v/v, lower phase). S, ginsenoside standards (PPD type ginsenoside mixtures).

**Figure 7:**
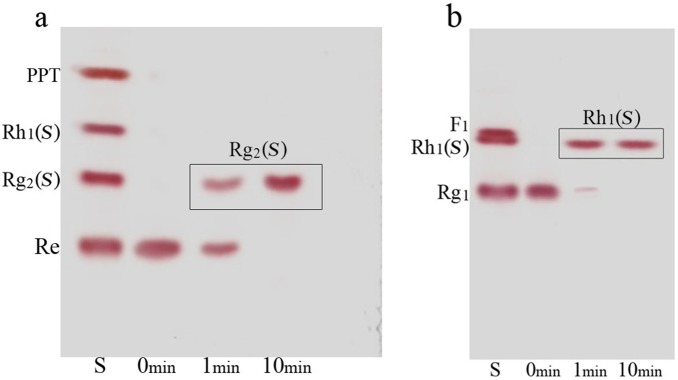
TLC analyses of time course of ginsenosides bioconversion by BglPC28. (a), transformation of ginsenoside Re; (b), transformation of ginsenoside Rg_1_. Developing solvent: CHCl_3_-CH_3_OH-H_2_O (65∶35∶10, v/v, lower phase). Lanes S, ginsenosidestandards (PPT type ginsenoside mixtures).

**Figure 8:**
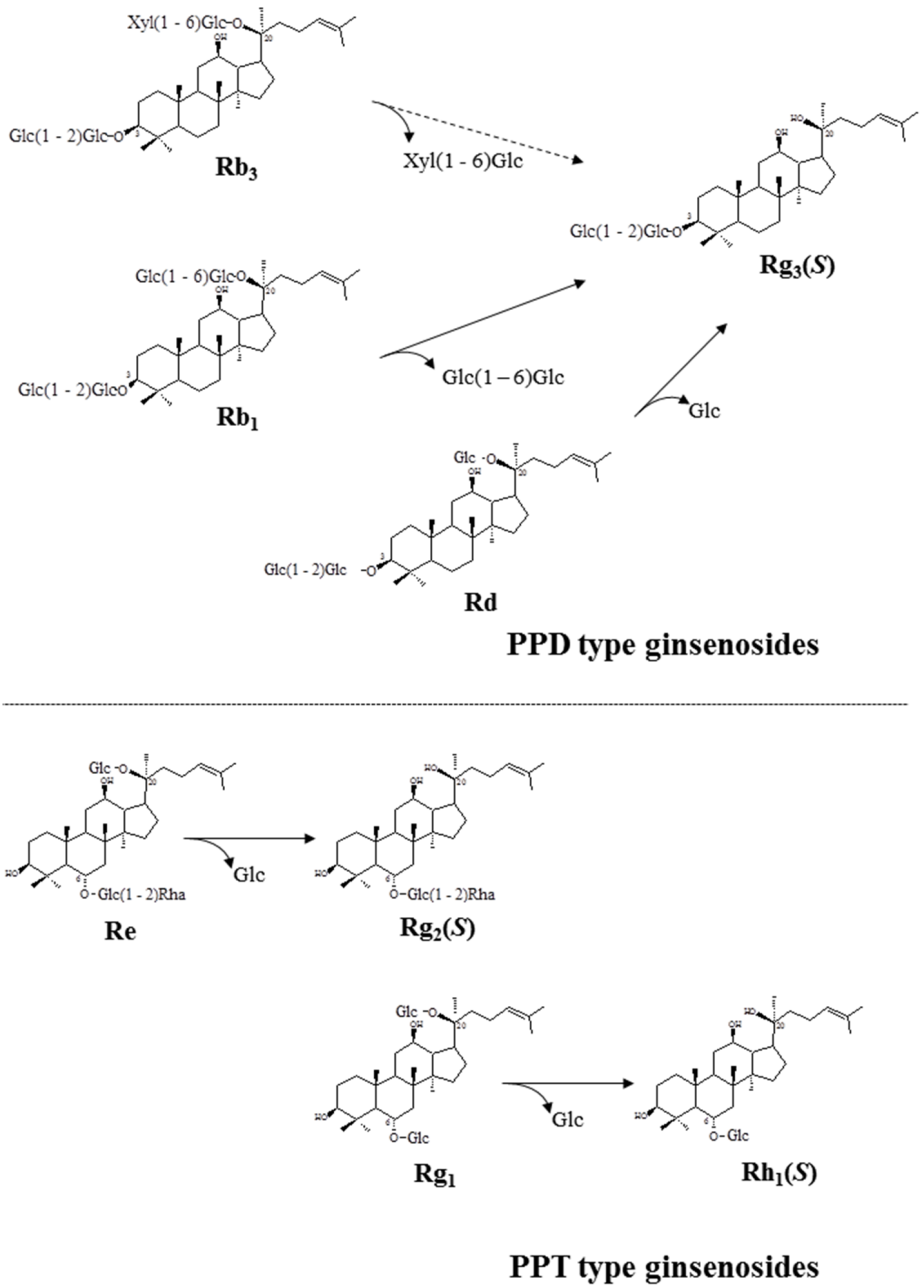
Transformation pathways of ginsenosides Rb_1_, Rb_3_, Rd, Re, and Rg_1_ by recombinant BglPC28, respectively to become biotransformed ginsenosides.

For verification of the bioconversion pathway of the five PPD type ginsenosides (Rb_1_, Rb_2_, Rb_3_, Rc, and Rd) and two PPT type ginsenosides (Re and Rg_1_) by GST-BglPC28, TLC and HPLC analyses were performed at regular intervals. GST-BglPC28 could clearly transform four major ginsenosides (Re, Rg_1_, Rb_1_, and Rd), as shown by the R_f_ values of the TLC analysis (Fig. 6) and the retention times in HPLC (data not shown). BglPC28 could efficiently hydrolyze the glucose moiety at the C20 position of Re and Rg_1_, transforming them into Rg_2_(*S*) and Rh_1_(*S*), respectively (Fig. 7a, 7b). In addition to these PPT-type ginsenosides, BglPC28 could also transform the PPD-type ginsenosides, Rb_1_, Rb_3_, and Rd. The proposed biotransformation pathways for the actions of BglPC28 on the PPD-type ginsenosides are Rb_1_ or Rd → Rg_3_(*S*) via hydrolysis of the outer glucoses directly at the C20 position. Furthermore, BglPC28 transformed Rb_3_ to Rd weakly, by hydrolyzing the D-xyloside moiety attached at the C20 site, as indicated by pNP-β-D-xylopyranoside activity in BglPC28 (Table 2). Overall, BglPC28 hydrolyzed Re, Rg_1_, Rb_1_, Rb_3_, and Rd as follows: Re → Rg_2_(*S*), Rg_1_ → Rh_1_(*S*), Rb_1_ → Rg_3_(*S*), Rd → Rg_3_(*S*), and Rb_3_ → Rg_3_(*S*) (Fig. 8).

The metabolic pathways of ginsenoside hydrolysis by BglPC28 are very similar to those of a β-glucosidase (Bgp1) from *Microbacterium esteraromaticum* [33], [34], and this is attributed to their high amino acid sequence similarity (Fig. 2). BglPC28 also has similar metabolic pathways of ginsenosides hydrolysis with a β-glucosidase (BglQM) from *Mucilaginibacter* sp. strain QM49 except that BglQM hydrolyzed Rb_2_ and Rc into C-Y and C-Mc, respectively, whereas BglPC28 did not hydrolyze them. In addition, the amino acid sequence similarity of both is very low and located in different subgroups (Fig. 2). Re and Rg_1_ were deglucosylated when exposed to a culture of *Pseudonocardia* sp. Gsoil 1536, and recombinant BglPC28 has the same effect, presumably via the hydrolyzing pathways. This suggests that hydrolyzing activity for ginsenosides Re and Rg_1_ of *Pseudonocardia* sp. Gsoil 1536 arises from the expression of BglPC28.

When Re, Rg_1_, Rb_1_, and Rd (1.0 mg/ml) were used as substrates, they were biotransformed within 10 min by 10 mg/ml of crude recombinant GST-BglPC28. Several other ginsenoside-hydrolyzing recombinant enzymes have been reported, but most were only able to hydrolyze the outer or inner glucose moiety at the C3 position of the aglycon [28], [31], [35], [36], [46], [53], [54], [55]. Three previously described enzymes, Bgp1 [35], BglAm [28] and BglQM [29], had the same metabolic pathways of ginsenoside hydrolysis for ginsenosides Re and Rg_1_ as BglPC28, but the authors conducted only a simple enzymatic characterization without further scale-up or process engineering and in some case low yield and a complicated purification step impeded practical application of these enzymes. Thus, we sought to optimize our BglPC28 reaction for industrially relevant 100 gram-scale production of Rg_2_(*S*).

### 3.7. Optimization of Re and Enzymes Concentration

**Figure 9:**
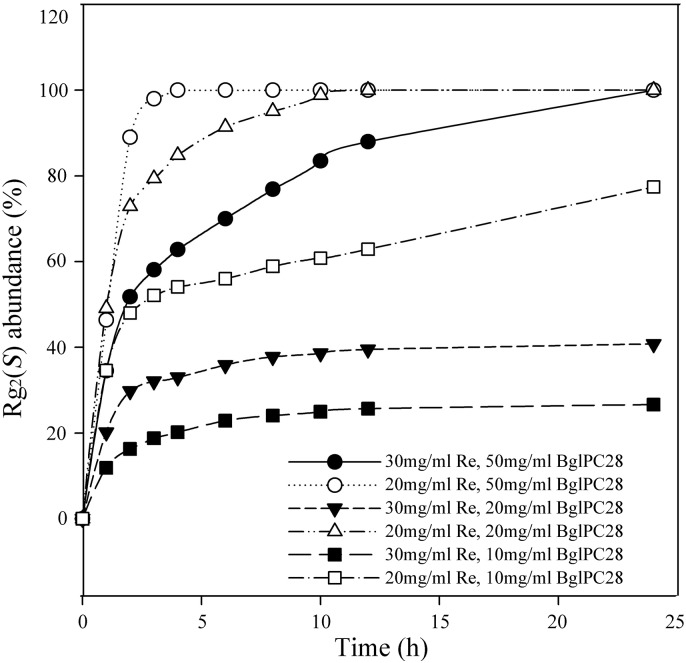
Effect of the concentration of Re and crude BglPC28 on the production of ginsenoside Rg_2_(*S*).

Two substrate concentrations (20 mg/ml and 30 mg/ml) and three crude enzyme concentrations (10, 20, and 50 mg/ml) as final concentrations were tested in order to determine the appropriate substrate concentration for decreasing the reactor volume and economical enzyme concentration to reduce production costs. The time course of the ginsenoside Re and product Rg_2_(*S*) was determined via HPLC analyses in six test conditions (Fig. 9). In the test conditions of a low substrate concentration (20 mg/ml) and a high crude enzyme concentration (50 mg/ml), the ginsenoside Re was completely converted to ginsenoside Rg_2_(*S*) within 4 hours; thisreaction speed is three times faster than that of the 20 mg/ml Re with 20 mg/ml crude enzyme concentration and six times faster than that of 30 mg/ml Re with 50 mg/ml crude enzyme concentration. Under the other three reaction conditions the conversion was not completed within 40 hours. Thus, these three reaction conditions were excluded in the next step. Providing the advantages of smaller usage of enzyme and complete conversion of Re, the conditions of 20 mg/ml substrate concentration and 20 mg/ml crude enzyme concentration were adopted for the next scaled-up biotransformation step.

### 3.8. Preparation of Crude pGEX- BglPC28 and Scaled-up Production of Ginsenosides Rg_2_(*S*)

**Figure 10:**
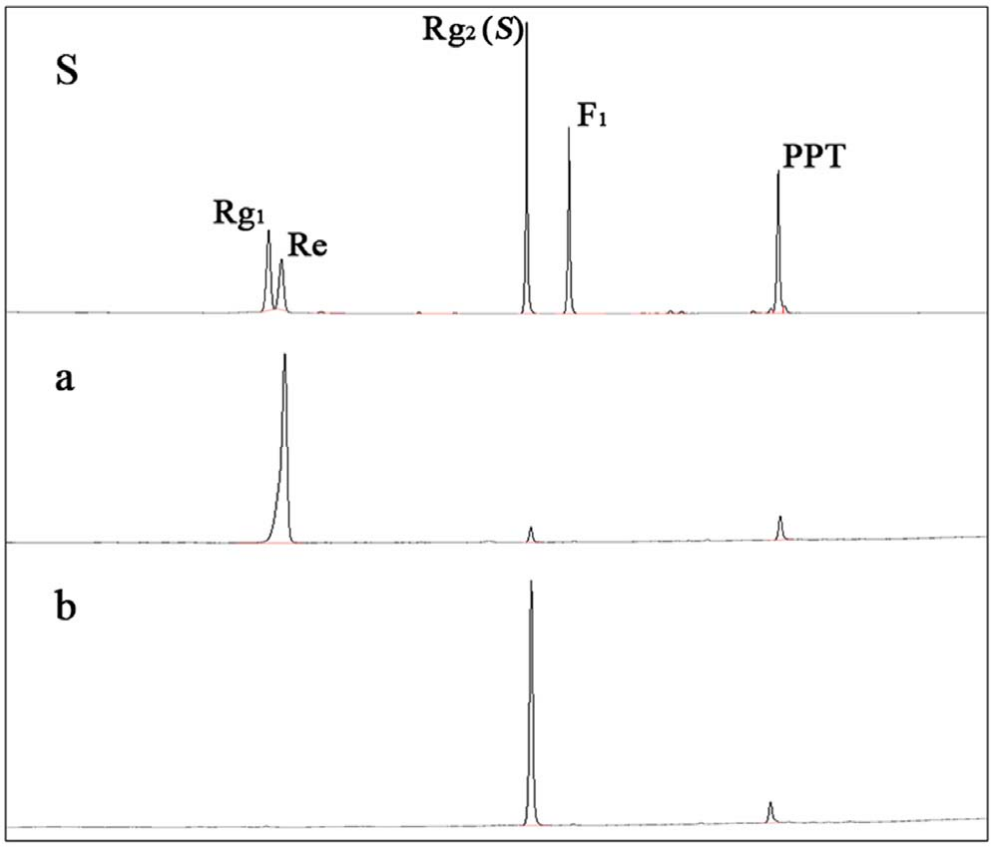
HPLC analysis of the transformation of Re by GST-BglPC28; S, standard ginsenosides; a, Re as substrate for production for Rg_2_(*S*); b, produced Rg_2_(*S*) after treating Re and GST-BglPC28 for 24 hours.

The bacterial cells that harbor pGEX-*bglPC28* and were incubated further for 18 h at 22°C after induction were harvested via centrifugation when the culture reached an OD of 38 at 600 nm. 154 g of wet cells was harvested and the pellets were resuspended in 10 volumes (w/v) of 100 mM sodium phosphate buffer (pH 7.0). The cells were disrupted via ultrasonication and the supernatant was used as crude enzymes for the biotransformation of the ginsenosides. As almost half of the expressed pGEX•BglPC28 was in soluble form, the crude recombinant BglPC28 was applied to the biotransformation reactor. The enzyme reaction occurred using the crude recombinant BglPC28 with RG-Re as the substrate with a concentration of 20 mg/ml, and crude recombinant BglPC28 was adjusted to 20 mg/ml as the final concentration in 7.5 L in order to produce Rg_2_(*S*). The ginsenoside Re was completely converted to Rg_2_(*S*) within 24 hours after the crude GST-BglPC28 was applied to the ginsenoside Re (Fig. 10). The research team behind this paper searched the ginsenoside hydrolyzing bacteria [27], [56], [57] and constructed several ginsenoside-hydrolyzing recombinant enzymes [28], [29], [30], [31], [46], [48], [58], [59] by surveying the ginsenoside-hydrolyzing enzymes. However, these enzymes are not applicable or are not active in transforming the Re. At last, the finding of exceptional BglPC28 that can efficiently convert Re is a key factor in creating Rg_2_(*S*).

### 3.9. Purification of Biotransformed Rg_2_(*S*)

**Figure 11:**
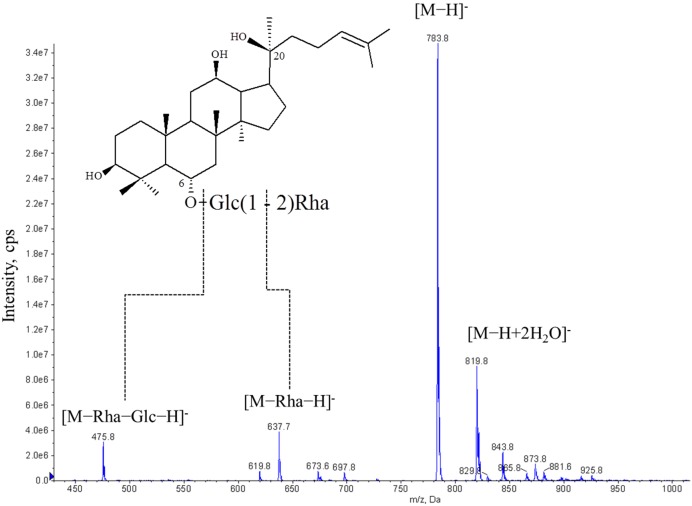
Electrospray negative ion mass spectrum of produced ginsenoside Rg_2_(*S*) of which m/z was 783.8 [M−H]^−^. Glc, glucose moiety; Rha, rhamnose moiety.

In order to remove the enzymes, salt, and free sugars from the reaction mixture of the 7.5 L reaction of Re with BglPC28, the mixture was centrifuged at 5,000 rpm for 15 min. Most of the ginsenoside Rg_2_(*S*) was precipitated to form a solid, with a small quantity remaining dissolved in the supernatant (data not shown). After a purification step using a column chromatograph packed with HP20 resin, approximately 24 L of the 95% ethanol eluent was evaporated *in vacuo* in order to create 113 g of ginsenoside Rg_2_(*S*). Its chromatographic purity was 84.0±1.1%, as determined via HPLC. Furthermore, electrospray negative ion mass spectrum of produced ginsenoside Rg_2_(*S*) was obtained on API 2000 to confirm its identity. Produced Rg_2_(*S*) was a white powder with a pseudomolecularion peak [M−H]^−^ at m/z 783.8 in ESIMS, corresponding to C_42_H_72_O_13_ (calculated molecular weight, 784.5). Thus, produced Rg_2_(*S*) was confirmed (Fig. 11). Among Re, the total molar amount of Re that could be biotransformed into Rg_2_(*S*) using BglPC28 was 138.8 mmol, which corresponds to 131.4 g of 150 g. The residue (18.6 g) was composed of other types of ginsenosides, moisture and unknown impurities. The molar amount of the produced ginsenoside Rg_2_(*S*) was 120.9 mmol. This indicates that the recovery ratio through the biotransformation process using ginsenoside Re to Rg_2_(*S*) reached 87.1% after bioprocess engineering.

## Discussion

Although Rg_2_(*S*) has neuroprotective, anti-inflammation, and anti-diabetic effects, the lack of a selective mass-production technology has hampered its commercial use. Red ginseng, which is a very popular health-promoting food in oriental herbal medicine, contains approximately less than 0.02% Rg_2_(*S*) based on dry weight. In total, red ginseng products account for 52.8% of the total health food market in Korea, which was estimated to be worth 625 million US dollars in 2011 [60]. Furthermore, one Korean cosmetic company (Somang Co. Ltd.) recently launched a brand name RGII product that contains the ginsenoside Rg_2_ as the major active component. However, the high cost of purified Rg_2_ from red ginseng obstructs its widespread use.

To achieve Rg_2_(*S*), a number of researchers have sought to biotransform major ginsenosides into larger quantities of Rg_2_(*S*) or Rh_1_(*S*) using microorganisms [61], [62] and enzymes [29], [35], [63], [64] in laboratory settings. Ko et al [63] characterized the abilities of crude β-galactosidase from *Aspergillus oryzae* and crude lactase from *Penicillium* sp. to biotransform PPT-type ginsenosides to Rg_2_(*S*) and Rh_1_(*S*), but only on a 10-mg scale. The usage of cloned ginsenoside-hydrolyzing GHs in glycoside hydrolase families 1, 3, and 51 are the other method. As shown in Table 4, family 3 GHs has various types of ginsenoside-hydrolyzing pathways. As such, they have strong potential for the production of various types of minor ginsenosides from major ginsenosides for practical application. Recently, three recombinant β-glucosidases belonging to glycoside hydrolase family 3 were shown to transform Re and Rg_1_ into Rg_2_(*S*) and Rh_1_(*S*), respectively. BgpA from *Microbacterium esteraromaticum* and BglAm from *Actinosynnemamirum*, but the level of research remains only at the level of simple enzyme characterization, and sufficient efficiency for gram-scale applications has not been obtained [28], [35]. The other (BglQM), from *Mucilaginibacter* sp. QM49, was applied to obtain a gram scale Rg_2_(*S*) and Rh_1_(*S*) using a PPT type ginsenoside mixture. However, the yield was low (33.4%) and the final purification step using silica resin was complicated. As a family 3GH, BglPC28 exhibited a similar ginsenoside-transforming pathway to those of BgpA and BglQM, but showed high conversion activity using Re, which was not easily dissolved in water. Re was readily dissolved in DMSO up to 200 mg/ml. Here, we report for the first time that BglPC28 can transform up to 20 mg/ml of Re into 100 gram-scale Rg_2_(*S*) within 24 h. Our novel identification of BglPC28 as an enzyme capable of converting Re and Rg_1_ into Rg_2_(*S*) and Rh_1_(*S*) is expected to facilitate mass production of Rg_2_(*S*) from protopanaxatriol-type ginsenoside Re derived from *Panax quinquefolius* (American ginseng) or *Panax ginseng* C. A. Meyer (Korean ginseng).

**Table 4:** Major ginsenosides transformations by the cloned glycoside hydrolases family 3.

Glycoside hydrolase name	Subfamily	Microorganism	Ginsenoside conversion pathway	Reference
BGL1	4	*Aspergillus niger*	Rf→Rh_1_(*S*)	[47]
			Rb_1_→Gyp XVII→Gyp LXXV	
			Rb_2_→C-O→C-Y	
BgpA	2	*Terrabacter ginsenosidimutans*	Rc→C-Mc_1_→C-Mc	[46], [65]
			Rd→F_2_→C-K	
			Rg_3_→Rh_2_	
			Rb_1_→Gyp XVII→Gyp LXXV→PPD	
			Rb_1_→Gyp XVII→Rh_2_(*S*)→PPD	
			Rb_2_→C-O→C-Y	
BglAm	2	*Actinosynnema mirum*	Rc→C-Mc_1_→C-Mc	[28]
			Rd→F_2_→Rh_2_(*S*)	
			Rg_3_(*S*)→Rh_2_(*S*)→PPD	
			Re→Rg_2_(*S*)	
			Rg_1_→Rh_1_(*S*)→PPT	
			Rb_1_→Gyp XVII→Gyp LXXV→C-K	
			Rb_2_→C-O→C-Y	
			Rc→C-Mc_1_→C-Mc	
BglSk	2	*Sanguibacter keddieii*	Rd→F_2_→C-K	[31]
			Rg_3_(*S*)→Rh_2_(*S*)→PPD	
			Re→Rg_2_(*S*) or F_1_	
			Rg_1_→F_1_	
			Rb_1_→Rd→Rg_3_(*S*)	
			Rb_2_→C-O→C-Y	
BglQM	2	*Mucilaginibacter* sp. QM49	Rc→C-Mc_1_→C-Mc	[29]
			Re→Rg_2_(*S*)	
			Rg_1_→Rh_1_(*S*)	
rApy-H1	5	*Bifidobacterium longum* H-1	Rb_2_→Rd	[45]
BglBX10	5	*Flavobacterium johnsoniae*	Rb_1_→Rd→Rg_3_(*S*)	[48]
			Rb_1_→Rg_3_(*S*)	
Bgp1	5	*Microbacterium esteraromaticum*	Rd→Rg_3_(*S*)	[35], [36]
			Rg_1_→Rh_1_(*S*)	
			Re→Rg_2_(*S*)	
			Rb_1_→Rd→Rg_3_(*S*)	
BglPC28	5	*Pseudonocardia* sp. Gsoil 1536	Rb_3_→Rg_3_(*S*)	This study
			Re→Rg_2_(*S*)	
			Rg_1_→Rh_1_(*S*)	

In summary, we herein describe the isolation of a ginsenoside-hydrolyzing β-glucosidase (BglPC28) belonging to glycoside hydrolase family 3 from *Pseudonocardia* sp. Gsoil 1536, and the production of a recombinant enzyme for the biotransformation of the major ginsenoside Re into the pharmacologically active rare ginsenoside Rg_2_(*S*). This enzyme was expressed in *E. coli* BL21(DE3) in a soluble form. Characterization revealed that its optimum reaction conditions were 37°C and pH 7.0, and that it could be used for 100 gram-scale Rg_2_(*S*) production. In terms of yield, 113 g of Rg_2_(*S*) with 84.0±1.1% chromatographic purity was obtained via the biotransformation of 150 g of a purified ginsenoside Re followed by purification with HP20 resin. Bioconversion process was taken place in a 10 L jar fermenter in 100 mM sodium phosphate buffer (pH 7.0) containing 10% DMSO at 30°C for 24 h, with an initial substrate concentration of 20 mg/ml. This is the first report of a cloned enzyme that is capable 100 gram-scale production of Rg_2_(*S*) through biotransformation of the major ginsenoside Re.
